# DNA Homeostasis and Senescence: Lessons from the Naked Mole Rat

**DOI:** 10.3390/ijms22116011

**Published:** 2021-06-02

**Authors:** Harvey Boughey, Mateusz Jurga, Sherif F. El-Khamisy

**Affiliations:** 1 The Healthy Lifespan Institute and the Institute of Neuroscience, University of Sheffield, Sheffield S10 2TN, UK; hboughey1@sheffield.ac.uk; 2 The Institute of Cancer Therapeutics, University of Bradford, Bradford BD7 1DP, UK; m.jurga@bradford.ac.uk

**Keywords:** naked mole rat, senescence, neurodegeneration, ageing, DNA damage, DNA repair, oxidative stress, reactive oxygen species

## Abstract

As we age, our bodies accrue damage in the form of DNA mutations. These mutations lead to the generation of sub-optimal proteins, resulting in inadequate cellular homeostasis and senescence. The build-up of senescent cells negatively affects the local cellular micro-environment and drives ageing associated disease, including neurodegeneration. Therefore, limiting the accumulation of DNA damage is essential for healthy neuronal populations. The naked mole rats (NMR) are from eastern Africa and can live for over three decades in chronically hypoxic environments. Despite their long lifespan, NMRs show little to no biological decline, neurodegeneration, or senescence. Here, we discuss molecular pathways and adaptations that NMRs employ to maintain genome integrity and combat the physiological and pathological decline in organismal function.

## 1. Introduction

An exposure to low oxygen conditions (hypoxia) perturbs oxidative phosphorylation, which in turn leads to an increase in cellular concentration of reactive oxygen species (ROS). ROS can cause oxidative stress, which is capable of ageing human cells through mutation induction and protein oxidation, potentially causing senescence and neurodegeneration [1,2,3,4]. Interestingly, naked mole rats (*Heterocephalus glaber*) (NMRs), which are mouse-sized rodents, live for 30 years inside of hypoxic burrows and show minimal senescence and age-related neurodegeneration [5,6,7,8]. Moreover, NMRs display a high prevalence of protein plaques (tau and amyloid β), which are currently believed to be a significant cause of neurodegeneration, but fail to exhibit any signs of neuronal death [6]. Therefore, understanding the NMR’s resilience to degeneration despite their environment or age might be greatly beneficial for healthcare. This review aims to summarise current knowledge on molecular pathways that NMRs employ to ensure genome integrity and minimal cellular senescence to achieve neuroprotection.

## 2. DNA Damage Response in NMRs

### 2.1. DNA Damage Response Upregulation

DNA homeostasis is the process of maintaining a faithful structure and optimum environment for DNA, which is key to a long and healthy lifespan [9]. Cells must ensure fidelity of DNA, as gradual mutational build-up has been linked to ageing and neurodegeneration [9]. Therefore, a variety of DNA damage response (DDR) pathways evolved to repair damaged DNA [10]. RNA-seq analysis showed a higher transcription rate of DDR-associated genes in human and NMR samples than in mouse [11]. Specifically, upon DNA damage induction, both NMR and human cells increased transcription of repair factors associated with non-homologous end joining (NHEJ), mismatch repair (MMR), homology-directed repair (HDR) and base excision repair (BER) [11]. Higher transcription rates of DDR genes triggered by DNA damage might ensure a large mRNA pool for DDR protein synthesis and subsequent DNA repair. Interestingly, BER genes in NMRs showed comparable transcription levels to the mouse, apart from 5 out of 11 DNA glycosylases, which were over-expressed. These glycosylases function to label damaged bases for BER. As a result, NMR BER resolved DNA damage more rapidly, showed improved efficiency of excision and faster return to basal transcription levels, when compared to the mouse [11,12,13]. Rapid labelling imparted by a large glycosylase pool shortened the period of genotoxicity and enabled a faster return to normal homeostasis post-damage [12,14]. Moreover, NMR p53 protein shows high expression and an extreme cellular half-life [15]. No reduction in p53 abundance was observed 8 h post-translation inhibition in NMRs, as compared to mice, where no signal was seen after 2 h [15]. That is important because cells with low p53 expression experience more significant DNA damage upon reoxygenation (reperfusion) [16], suggesting that high p53 levels are key to survival in hypoxia. In humans and NMRs, increased DDR gene transcription and efficient DNA repair correlates with a longer life span [11]. Extended periods of genotoxicity can induce formation of prevalent double-stranded DNA breaks (DSB), which can trigger senescence and apoptosis [17]. By accelerating the rate of DNA repair, excessive damage signals are less likely to occur, thereby reducing the chances of a senescent-cell build-up, which is a hallmark of a number of ageing associated disease, such as cancer and neurodegeneration [4].

Microhomology-mediated end joining (MMEJ) is an error-prone repair pathway, which requires both PARP1 and XRCC1 and is capable of causing large genome rearrangements, potentially triggering neurodegeneration and senescence [18,19,20]. XRCC1 acts as a molecular scaffold, binding repair proteins at damaged regions, while PARP1 labels damaged DNA for recruitment of DDR factors [21,22]. Notably, upon UV-induced damage in NMRs, steady-state XRCC1 and PARP1 mRNA levels diverged [12]. PARP1 mRNA increased, whilst XRCC1 mRNA decreased. Reduction in XRCC1 expression might inhibit highly error-prone MMEJ [19,23] and redirect the repair efforts towards less error-prone NHEJ pathways, whose genes are highly transcribed in NMRs [24]. Moreover, high PARP1 expression was previously correlated with lower recombination rates [23], which in turn might protect from chromosomal rearrangements, oncogene activation, or development of Alzheimer’s disease [25].

### 2.2. Gene and Protein Adaptations

NMRs evolved copy number and structure adaptations of DDR genes to augment their DNA homeostatic ability. The NMR has three copies of CCAAT enhancer binding protein gamma (CEBPG) [13]—a transcription factor (TF) that activates antioxidant and DNA damage repair genes. Increased copy number of CEBPG may be implicit in higher basal levels of antioxidants [26,27], which in turn safeguard cells from reactive oxygen species and correlate with greater neuroprotection [28]. CEBPG has also been shown to supress senescence [29]. Notably, NMR genome contains the *RPA4* gene, which was previously seen only in primates and horses [30]. The canonical replication protein A (RPA), which is a complex of RPA1, 2 and 3, has a high affinity with ssDNA. It binds ssDNA during replication and DNA repair, preventing secondary structure formation [31]. *RPA4* is an analogue of *RPA2*, which forms a noncanonical RPA complex (containing RPA4) that shows an increased affinity wih DNA damage factors and facilitates cell cycle stalling [32]. Therefore, RPA4 function may allow NMR cells to conduct swift and accurate DNA repair without risking SSB to DSB conversion.

### 2.3. Antioxidants for DNA Protection

The ability to prevent DNA damage before it happens is better than simply repairing it. CEBPG (with an extra gene copy in NMRs) is implicit in the antioxidant response [13]. CEBPG promotes transcription of antioxidant genes, including superoxide dismutase 1 (SOD1) and glutathione S-transferases (GST) [27,30]. SOD1 functions to remove superoxide radicals from the mitochondria [33,34], while GST conjugates glutathione (an antioxidant) to target xenobiotic molecules for their excretion [35]. SOD1 activity, which is dependent on SOD1 binding to zinc ions [36,37], worsens over time as zinc homeostasis is gradually lost in ageing humans [38]. Alpha 2 Macroglobin (A2M) binds zinc and functions as a protease inhibitor and chaperone against protein aggregation [39]. NMRs contain 140X more A2M in their livers and 2–3X more in the blood than mice do [40]. High A2M expression in the blood may be binding and supplying zinc to cells for detoxifying enzymes, such as those having their transcription stimulated by CEBPG (e.g., SOD1). This might explain NMRs’ resistance to zinc toxicity [41] and optimal zinc homeostasis throughout their lives, which might promote efficient SOD1 activity. To further strengthen the importance of active SOD1, its loss of function is related to the neurodegenerative disorder amyotrophic lateral sclerosis (ALS) [37]. In mice, SOD1 knockout reduced the lifespan by 30% [42]. Optimum function imparted by better zinc homeostasis, via high A2M levels, may be protecting NMRs from neurodegeneration during hypoxic conditions, which would typically trigger or exacerbate DNA damage. NMRs’ extra *CEBPG* copy may not only be policing the genome for damage, but may also be implicit in the NMR effective antioxidant response, known to be key to long life spans [43].

Nuclear factor erythroid 2-related factor 2 (NRF2) is a TF, activating over 200 genes associated with the antioxidant and anti-inflammatory response, under constant activity in NMRs [44]. NRF2 high activity is regulated at the transcriptional and proteasomal level [44]. 2.5X higher transcription of *NRF2* is seen in NMRs as compared to mice; KEAP1 E3 ligase, which negatively regulates NRF2 via ubiquitination, is 3X less transcribed in NMRs than mice [44]. BRCA1, also implicit in stability and activity of NRF2, shows positive selection in the protein binding region interacting with NRF2 [45]. Positive selection in a region of BRCA1 intrinsic to NRF2 stability and reduced KEAP1-dependent NRF2 degradation suggests a selective advantage for constitutive high antioxidant gene expression in NMRs. Together, these findings highlight the importance of NRF2 function in the unique fitness of the NMR [28].

### 2.4. Epigenetic Stability

Epigenetic marks are chemical groups added to DNA or histones, which modulate DNA accessibility and transcriptional state [46]. Loss of these marks can cause reactivation of proliferative genes, which might deregulate metabolism due to loss of tissue-specific gene expression—characteristic of neurodegeneration, ageing and cancer [47,48]. Epigenetic rewriting is also central to induced pluripotent stem cells (iPSC) production, the class of pluripotent cells generated from differentiated somatic cells [49]. The NMR shows resistance to epigenetic rewriting. Initial, canonical iPSC induction proved unsuccessful; NMR cells required a knockdown of retinoblastoma protein (Rb) before successful iPSC induction could occur [50]. Rb is a tumour suppressor implicit in maintaining a repressive chromatin state at key pluripotency-related genes, with its loss triggering a weak activation at these loci [51,52]. This suggests that NMR genome is highly compact since the Rb knockdown potentially triggered more open chromatin states, which in turn eased IPSC induction. Moreover, many pluripotency related genes in mice contain bivalent domains and their chromatin landscape is euchromatic [50,53]. Bivalent chromatin regions contain both activating and repressing marks, with these loci poised for complete activation or repression [50,54]. On the other hand, NMR cells lack this bivalence; their chromatin landscape shows extremes of compaction state [50], which restricts epigenetic writers access to heterochromatic loci [55]. Though NMRs’ exact mechanism of epigenetic stability is still being researched, the NMR’s lack of bivalent domains and inferred Rb-driven chromatin compaction might synergise to create a severely restrictive chromatin landscape, efficiently excluding iPSC induction factors and maintaining a faithful epigenome throughout their life [50,52]. The gradual loss of epigenetic marks in human cells contributes to ageing phenotypes, such as poor hair growth and slowed collagen production [56]. NMRs resistance to iPSC induction may indicate a faithful epigenetic signature throughout their lives, accounting for their minimal age-related adverse phenotypes.

## 3. Preventing and Avoiding Senescence

### 3.1. Senescence-Associated Secretory Phenotypes

Senescence is the process of a cell cycle arrest triggered by multiple causes, including excessive DNA damage. Senescent cells undergo metabolic changes, including restructuring their secretome, which is a sum population of all secreted factors [17]. The senescent secretome contains pro-inflammatory molecules, proliferative signals and proteases, which all together are named senescence-associated secretory phenotypes (SASP) [17]. SASP can block the clearance of senescent cells leading to their accumulation, which is a hallmark of age-related diseases (e.g., Alzheimer’s disease, Parkinson’s disease) [4,57,58]. Research into drugs called senolytics, which help clear senescent cells from the body, has shown great promise in reducing age-related phenotypes. Mice treated with senolytics increased their lifespan by 30% and reversed age-related organ deterioration [59]. Moreover, the removal of senescent cells reduces SASP accumulation, which helps to return to optimum tissue function [60]. Notably, the NMR shows a low prevalence of senescent cells; this correlates positively with longevity [61,62].

### 3.2. Avoiding Senescence in Hypoxia

Neurons can exhibit senescent-like phenotypes impairing local tissue [63]. NMR neurones are often exposed to hypoxia, due to their low oxygen habitat, which has been shown to be toxic to human neurons [64]. Thus, resistance of NMR neurons to functional decline and senescence-like phenotypes upon hypoxia is essential to NMRs’ longevity. Compared to mice, NMR neurones showed 4X increased survival rates 2 weeks post mechanical damage and expressed STAT3, which was absent in control mice. STAT3 is a transcription factor that receives cell surface signals from cytokines and growth factors and drives neuronal repair [65,66]. Furthermore, post-damage NMR neurones had low caspase-3 steady state levels [66], which is a protein driving apoptosis [67].

Schwann cells are wrapped around axons to generate the myelin sheath [68] and support axonal regeneration [69]. However, their repair capacity after initial damage gradually decreases over time [70]. Interestingly, sustained expression of STAT3 resulted in the retention of Schwann cells’ regenerative capacity in humans [71]. As NMR cells maintain STAT3 expression, they presumably do not lose the capacity for axon regeneration. Moreover, due to elevated A2M levels, NMR neurones maintain optimal zinc homeostasis, which is required for myelination—a crucial step in axon regeneration [39,40,72]. Finally, NMRs’ low caspase-3 signal suggests weak apoptotic response to neuronal damage, that might suggest normal brain development and continual neurogenesis [73].

Another key component of the nervous system is glia cells. Astrocytes are star-shaped cells, found in the brain and spinal cord with various functions [74]; they support endothelial cells responsible for the blood brain barrier, shuttle nutrients to neurones and bolster neuronal repair. Notably, NMRs show very low levels of astrocyte senescence [66]. Astrocytes have a high glycolytic rate [75] and the presence of astrocytes improves neuronal recovery by inducing neurogenesis and angiogenesis, as ablation of astrocytes in mice inhibited neuronal repair [76,77]. NMRs preferentially use fructose for glycolysis throughout their body during hypoxic conditions. Direct phosphorylation of fructose by keto-hexokinase (KHK) (producing fructose-1-phosphate, F1P) allows its entry into glycolysis [78]. As KHK and F1P accumulates in NMR brain during hypoxia, glycolysis is promoted regardless of the tissues energy status [78]. Moreover, lactate can be produced from pyruvate—the end product of glycolysis. Lactate is a crucial energy source for neurones after being shuttled to them from astrocytes. Lactate is converted by neurones back to pyruvate for entry into the Krebs cycle [79]. Preferential F1P-driven glycolysis in NMR astrocytes may not only be used to support NMR brain function, but it also produces lactate that fuels oxidative phosphorylation during hypoxia.

### 3.3. Avoiding ER Stress-Induced Senescence

Proteasome is a protein complex that degrades unnecessary and damaged proteins (proteolysis) and recycles amino acids [80]. Proteasome inhibition by exogenous chemicals or stress can lead to a build-up of biologically dysfunctional proteins and reduce the pool of available amino acids [81]. This can prevent translation and induce endoplasmic reticulum (ER) stress, due to the accumulation of partially translated and misfolded proteins [82]. Subsequently, ER stress can induce senescence and apoptosis [17]. However, stress can be reduced by the protein unfolded response (PUR) and autophagy [83]. NMR 28S proteasome shows significant resistance to inhibition by exogenous chemicals as compared to mice. This is thought to be due to the novel heat shock protein 40/72 heterodimer (HSP40/HSP72) [84]. HSPs are stress-induced proteins acting as chaperones, ensuring correct protein structure or shuttling misfolded/damaged proteins for degradation [85]. Notably, HSP40/72 was found to interact with the NMR 28S proteasome and rescued other species’ cytosolic protein fractions from proteasomal inhibition [84]. NMRs also show much higher basal autophagy levels than mice [26] and require greater accumulation of misfolded proteins to trigger PUR [83]. Therefore HSP40/HSP72 may function as the primary chaperone during mild stress, while proteolysis and autophagy may replenish the pool of amino acids, which all together reduces ER stress and inhibits PUR response [86]. This is beneficial as excessive PUR in the ER can trigger senescence [17].

The 28S rRNA is the structural RNA found in the ribosomes’ large subunits and is vital in decoding mRNA for translation. NMR 28S contains a novel fragment, originating from a transposable element [87], which, once excised, splits the 28S rRNA into two fragments. This is thought to create a more optimum ribosome architecture, since NMRs’ translational rate was comparable to mice’s, but the fidelity was determined to be 4–10X that of mice [87]. Crucially, high translational accuracy might extend cell viability. The inaccurate translation is likely to lead to sub-optimal enzymatic and protein–protein interactions, leading to accumulation of DNA damage, ER stress, PUR response and, subsequently, senescence [17,88]. Furthermore, hypoxia diminishes the ATP pool by perturbing oxidative phosphorylation, which subsequently compromises synthesis of aminoacylated tRNAs for translation, resulting in ER stress and PUR response [89]. NMR hypoxia-inducible factor 1 alpha (HIF1α) is the master regulator of the hypoxic response [90]. During hypoxia, HIF1α functions as a transcription factor triggering angiogenesis and metabolic compensation [90,91], whereas, during normoxia, it is bound by VHL for ubiquitin-dependent degradation [92]. Sequence analysis of NMR HIF1α and VHL shed light on unique changes to their sequences. The changes lead to a reduction in the degradation rates of HIF1α through a reduced affinity for VHL [93]. In most human cells, HIF1α is expressed constitutively at low levels, though usually degraded [94]. Due to their species-specific changes affecting HIF1α degradation, NMRs are likely to retain an active HIF1α pool to abrogate the effects of mild hypoxia. As a result, NMRs presumably require minimal time to acclimatise to fluctuating oxygen levels, reducing the window of cellular stress and avoiding stress-induced senescence.

As NMRs age, their ubiquitin-independent degradation, facilitated by 20S proteasome and ubiquitin-dependent degradation, facilitated by 26S proteasome, show a 1.5X increase in activity compared to young NMRs [95,96,97]. Moreover, age-related rise in expression of heat shock proteins (HSP) and ubiquitin ligases (UBE1, UBE2v2) was observed in NMRs [98]. UBE1 catalyses the first step of ubiquitin conjugation [99], labelling proteins to be chaperoned to the proteasome, whilst UBE2v2 has roles in stimulating and directly adding K63-ubiquitin chains to histone tails. K63-polyubiquitinated histones enhance the recruitment of DDR factors [100]. Higher basal levels of heat shock proteins as NMRs age might maintain high protein flux through the proteasome, preventing build-up of dysfunctional proteins [101]. Moreover, the increased activity of NMR ubiquitin-independent peptidase might remove unfolded/unstructured proteins arising from stress or mistranslation [102]. Therefore, proteasomal chaperone upregulation as NMRs age might increase flux through the proteasome (1.5X) to combat the age-associated build-up of dysfunctional proteins and key DDR proteins might abrogate the build-up of mutations.

The cysteine content in NMR proteome appears to be much higher than human or mice [103]. The amino acid cysteine contains a reactive thiol group and is usually clustered around enzyme active sites, leading to inactivation upon oxidation [104]. However, NMRs show high amounts of cysteine in non-functional protein regions too [26,103]. High accessory cysteine prevalence in NMR proteome may help buffer the cytoplasmic fraction against oxidation through ROS attenuation. Cysteine-driven attenuation of ROS may be synergising with CEBPG and NRF2-target genes. The NMR shows far higher retention of protein activity during high ROS conditions, potentially through accessory cysteine oxidation, and no age-related increase in their overall cysteine oxidation [103], which indicates efficient repair of those oxidised protein moieties, or removal of the excessively damaged proteins [105]. Accumulation of proteomic damage could trigger cellular senescence through ER stress, potentially explaining NMRs’ low prevalence of senescent cells.

### 3.4. Senescence Cell Death and Unique Senescence

NMRs exhibit the unique phenotype of senescent cell death (SCD) [106], causing senesced cells to spontaneously die, unlike other species where they must be removed directly by the immune system [17]. Immune-independent apoptosis of senescent cells in NMRs prevents build-up of toxic SASP. Upon artificial induction of senescence, NMR cells exhibited dysregulation of autophagy, ROS-sensitisation and spontaneous SCD [106]. Healthy NMR cells have a high autophagic response, antioxidant activity and mitochondria containing a high concentration of antioxidants and detoxifying enzymes [103,107]. The decrease in cellular recycling of biomolecules and elevated ROS upon hypoxia in NMR senescent cells may lead to the SCD phenotype. Thus, NMRs’ hypoxic environment may be working in their favour to maintain biological health through aiding senescent cell clearance.

NMR senescent cells transcriptome showed an expected increase in transcription of senescence-associated secretory phenotype (SASP) genes, but downregulation of genes in key pathways relating to SASP production and excretion [106]. NMR translation and ribosomal protein genes all demonstrated reduced transcription rates. Thus, when stress or DNA damage is sufficiently high, NMR cells are still able to enter senescence [108]. However, reduction in protein production and secretion results in lower production of SASP, protecting NMRs from their effects; NMR SCD phenotype enables effective clearance of senescent cells, abrogating their impact on the cellular microenvironment [106].

## 4. Conclusions

**Figure 1:**
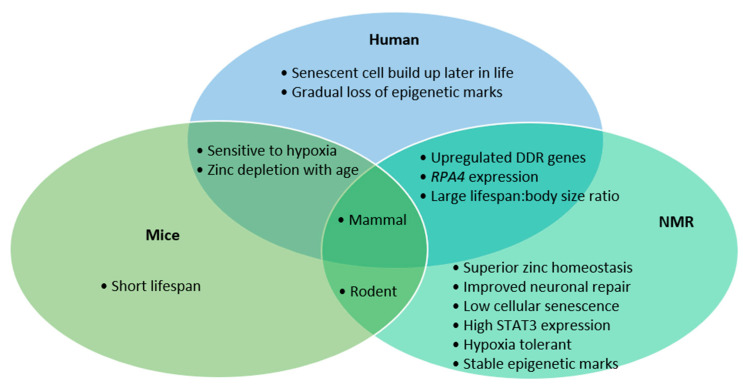
Summary of features relevant to neurodegeneration among mice, humans and naked mole rats. While mice and human cells are sensitive to hypoxia and lose zinc homeostasis with age, human and NMR cells share upregulated DNA damage response (DDR), including highly expressed mismatch repair, non-homologous end-joining, homology-directed repair and base excision repair pathways. Moreover, both NMRs and humans share a high lifespan to body ratio and express a unique subunit of the RPA complex—RPA4.

The NMR shows incredible resistance to various stressors and age-related decline. The similarities between humans and NMRs in DDR gene transcription, *RPA4* presence and their lifespan compared to body-size ratio makes them perhaps a more representative model than mice (Figure 1). However, the long-life span that makes NMRs so interesting also creates a hurdle to research, due to the studies’ longitudinal nature. The NMR’s exceptional neuronal preservation is the culmination of various mechanisms, namely improved antioxidant response, higher fidelity translation, stringent DNA repair, faithful proteome function, high functioning proteasome and low prevalence of senescent cells. These phenotypes are likely the result of evolution in harsh environments, leading to stringent homeostatic maintenance. What eventually causes their death would be an exciting research topic. Though few organisms can continue indefinitely, which processes show an increased burden as NMRs age may identify novel biological targets to mitigate our own degeneration.

## Data Availability

Not applicable.

## Abbreviations

DDR	DNA Damage Response
DSB	Double Strand Break
HDR	Homology-Directed Repair
MMEJ	Microhomology-Mediated End Joining
MMR	Mismatch Repair
NHEJ	Non-Homologous End-Joining
NMR	Naked Mole Rat
PUR	Protein Unfolded Response
ROS	Reactive Oxygen Species
SASP	Senescence-Associated Secretory Phenotypes
SCD	Senescent Cell Death
